# Enabling Global Access to Potent Subunit Vaccines with a Simple and Scalable Injectable Hydrogel Platform

**DOI:** 10.1039/d5bm01131k

**Published:** 2026-01-06

**Authors:** Priya Ganesh, Alexander N. Prossnitz, Carolyn K. Jons, Noah Eckman, Alakesh Alakesh, Ye Eun Song, Samya Sen, Eric A. Appel

**Affiliations:** a. Department of Materials Science & Engineering, Stanford University, Stanford, CA, USA.; b. Department of Chemical Engineering, Stanford, University, Stanford, CA, USA.; c. Department of Bioengineering, Stanford University, Stanford, CA, USA.; d. Sarafan ChEM-H Institute, Stanford University, Stanford, CA, USA.; e. Department of Pediatrics – Endocrinology, Stanford University, Stanford, CA, USA.; f. Woods Institute for the Environment, Stanford University, Stanford, CA, USA.

## Abstract

Vaccines have been crucial to dramatic improvements in global health in recent decades, yet next-generation vaccine technologies remain out of reach for much of the world. In particular, there are two overarching global needs: (i) develop vaccines eliciting more potent and durable immune responses, especially to reduce incidence of highly communicable diseases, and (ii) enable simple and cost-efficient formulation to maximize global access. Here, we develop an injectable hydrogel depot technology prepared through physical mixing of commercially available, generally recognized as safe (GRAS) polymers that can be formulated with subunit vaccine components to improve immune responses compared to standard vaccine formulations. We demonstrate that these hydrogels are shear-thinning and rapidly self-healing, enabling facile injection via injection, and they exhibit high yield stresses required for robust *in vivo* depot formation post-injection. These rheological properties proloong release of subunit vaccine cargo over a period of weeks, both *in vitro* and *in vivo*, and synchronize release kinetics across physicochemically distinct vaccine components (antigens and adjuvants). When used for formulation against wild-type SARS-CoV-2 and H5N1 influenza, these hydrogels enhance potency and durability of immune responses. This vaccine formulation technology can improve protection against current and potential future pandemic pathogens.

## 1. Introduction

Over the last century, vaccines have made great progress in lowering the incidence of several life-threatening infectious diseases such as smallpox, measles, tetanus, and polio.^1,2^ Unfortunately, poor vaccine coverage in low- and middle-income countries (LMICs) has prevented many of these diseases from being completely eradicated.^3^ Many vaccines are administered across multiple doses over several weeks or months to improve the potency and durability of the resulting immune response.^4^ These multiple-dose regimens, while effective, can drastically reduce access in LMICs due to high procurement and delivery costs.^5^ In addition, many vaccines, including those against SARS-CoV-2 and influenza, give way to breakthrough infections, leading to further spread of the disease as well as reduced confidence and willingness to receive further doses of the vaccine.^6–8^ Thus, there is a great need for single- or double-dose vaccines that are more potent and durable than current vaccines.

**Figure 1:**
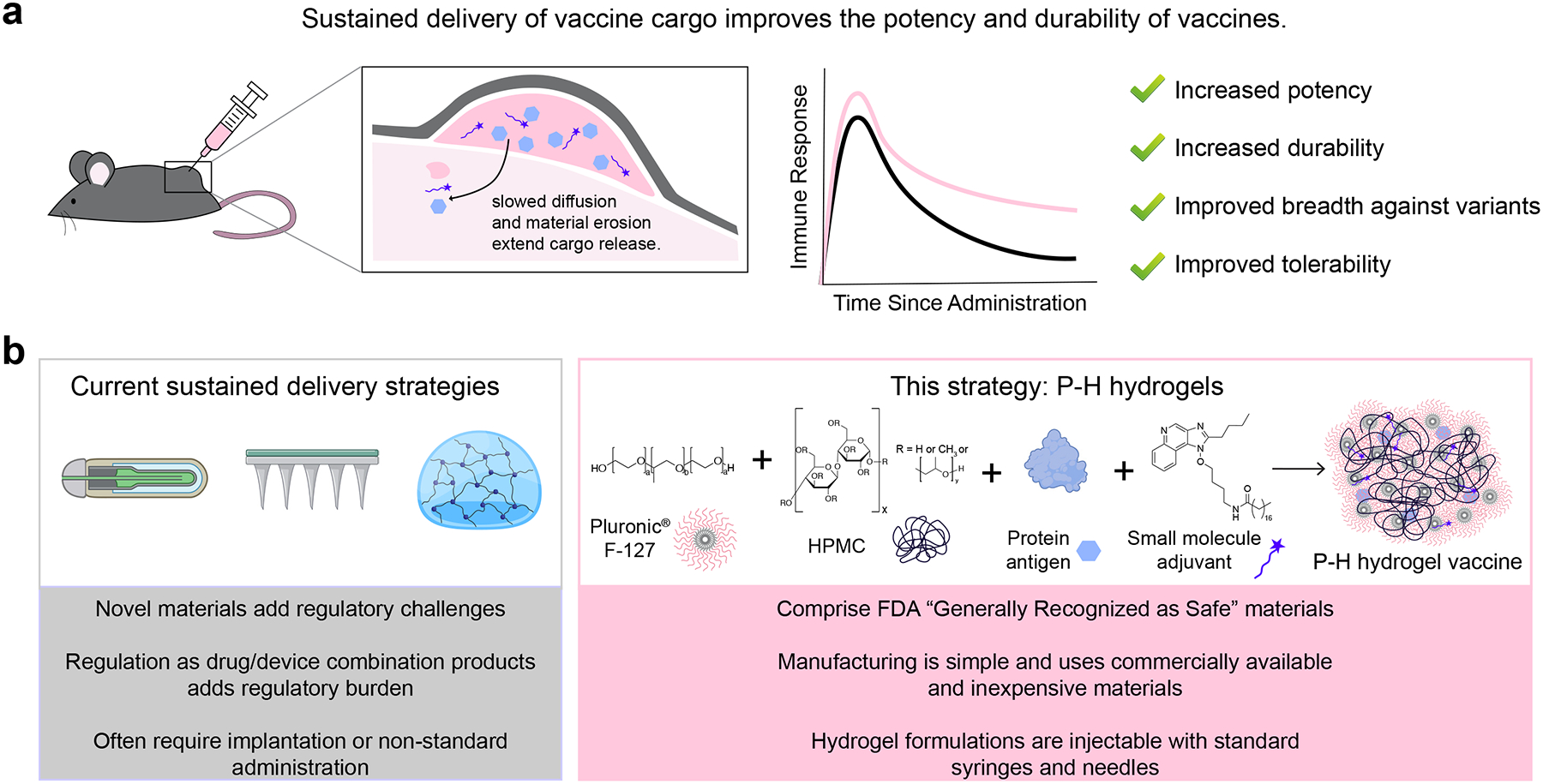
P-H hydrogels offer an easily scalable and translatable route for sustained delivery of vaccines. a) Sustained delivery of vaccine cargo leads to improved immune responses. B) P-H hydrogels comprised of physical blends of Pluronic^®^ F-127 and hydroxypropyl methylcellulose (HPMC) provide a scalable alternative to current delivery approaches for vaccines.

Sustained delivery technologies that release vaccine cargo over prolonged time-frames from 2–4 weeks have shown promise to meet this need. Single- or double-dose versions of these strategies include implantable osmotic pumps, microneedle patches, or injectable hydrogels.^9–15^ These strategies have been shown to improve the magnitude and durability of antibody titer responses to vaccines for a range of infectious diseases, including human immunodeficiency virus (HIV), various strains of influenza, and SARS-CoV-2 (Fig. 1a).^9,16–19^ Unfortunately, accessibility, scalability, and manufacturing concerns remain with many of these technologies. Osmotic pumps require surgical implantation, which would further exacerbate administration costs and reduce compliance. Microneedle patchbased vaccines can exhibit highly variable immune responses personto-person and local administration site reactions. In contrast, injectable hydrogels can be administered through standard administration routes, such as subcutaneous or intramuscular injections, and can be formulated through simple admixing with commercially available vaccines.^20–22^

We previously developed a self-assembled, injectable polymer-nanoparticle (PNP) hydrogel platform^23^ for a variety of biomedical applications (e.g., sustained biopharmaceutical delivery,^24,25^ adhesion prevention,^26^ and adoptive cell delivery^27^) that limits diffusional release of molecular cargo, enabling prolonged delivery over weeks to months. Other dynamic hydrogels, including supramolecular peptide-based hydrogels,^28^ hydrogels with dynamic covalent crosslinks,^29^ and *in situ* gelling systems^30^ have also exhibited promise for extended drug delivery. The dynamic nature of these crosslinking interactions imparts shear-thinning behaviors that enable facile administration by injection.^31,32^ In addition, these hydrogels rapidly self-heal following injection and exhibit high yield stresses, driving rapid formation of persistent depots following administration that enable prolonged cargo release.^33,34^ Further, these types of depot technologies have been shown to form transient inflammatory niches that can improve vaccine outcomes.^13,35^

While injectable hydrogels demonstrate promise as vaccine delivery vehicles, their preparation methods often require complicated chemical synthesis procedures and/or chemical modification of commercially available components, necessitating access to sophisticated chemical laboratory equipment. Moreover, generation of any “New Chemical Entity” according to the FDA increases regulatory requirements. To improve global access to sustained delivery technologies, creating a synthesis-free injectable hydrogel platform containing only GRAS listed components that meets the conditions necessary for sustained vaccine release remains a critical challenge.

To address this goal, we sought to design a hydrogel vaccine delivery system based on the commercially available amphiphilic polymer Pluronic^®^ F-127 (PF-127), which carries the “Generally Recognized as Safe” (GRAS) designation from the Food and Drug Administration (FDA).^36^ When dissolved in water, PF-127 self-assembles into a micellar network at elevated temperatures, where the sol-gel transition temperature is dependent on the polymer concentration.^37^ Yet, under aqueous sink conditions, such as the subcutaneous space *in vivo*, PF-127-based hydrogels tend to erode quickly, leading to accelerated loss of cargo despite a small mesh size.^38^ To overcome this challenge, PF-127 can be physically mixed with cellulose-derived polymers, which has been shown *in vitro* to limit gel erosion and improve cargo retention.^39^

In this work, we engineer a synthesis-free injectable hydrogel vaccine platform that we denote P-H hydrogels through physical mixing of dissolved PF-127, hydroxypropyl methylcellulose (which also carries the GRAS designation), and soluble subunit vaccine components, including protein antigens and small molecule adjuvants. Notably, these hydrogels are facile to prepare and have the potential to be translated quickly on account of their low-cost and commercially available constituents that are already part of numerous licensed drug products (Fig. 1b). We believe that the components of P-H gels will be regulated as excipients in the vaccine formulation, thus providing a much simpler regulatory path than a combination product.

We characterize the mechanical properties of P-H hydrogel formulations through shear rheology and injection force measurements to ensure that they have the required mechanical properties for injectable, depot-forming hydrogels. We then evaluate release kinetics of model vaccine cargo both *in vitro* and *in vivo* to determine the ability of P-H hydrogels to extend antigen delivery. Finally, we compare the P-H hydrogel platform to current liquid bolus approaches to formulate vaccines against wild-type SARS-CoV-2 and avian influenza A (H5N1). As of 2025, SARS-CoV-2 has claimed the lives of over 7 million people worldwide, and variants of the disease continue to emerge in areas of the world with low rates of booster shots.^40,41^ Recently, there have been several outbreaks in humans of the highly pathogenic H5N1 influenza, and while there has been no reported human-to-human spread, the disease exhibits an extremely high mortality rate and the potential to mutate to become more easily transmissible. Thus, we demonstrate the ability of the P-H hydrogel platform to enhance the potency and durability of vaccines against these current or potential future pandemic pathogens.

## 2. Results and Discussion

### 2.1. Preparation and Rheological Characterization of P-H Hydrogels

**Figure 2:**
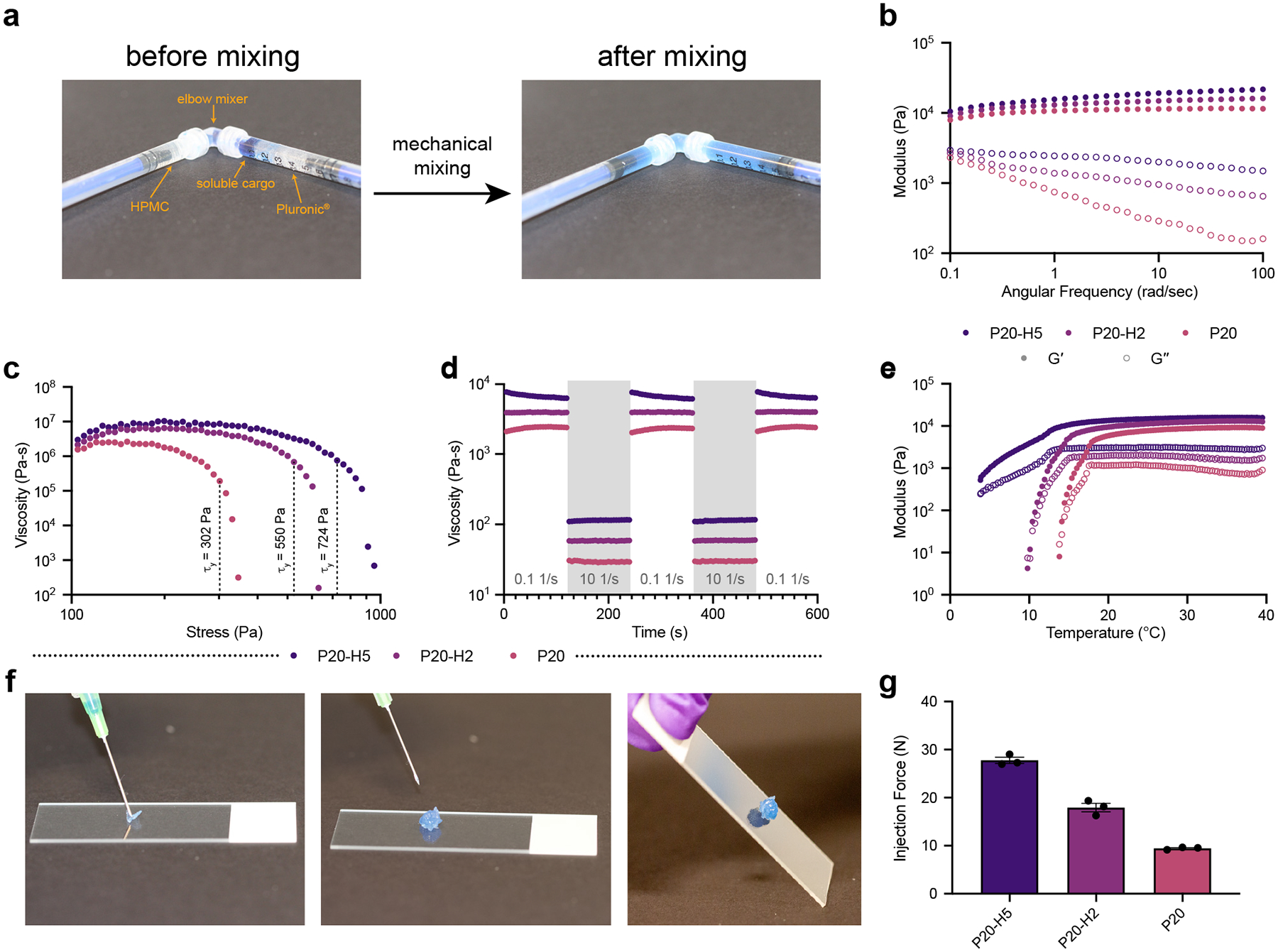
Preparation and rheological properties of P-H hydrogels. a) Loading and mixing of PF-127, HPMC, and soluble components into a homogeneous P-H hydrogel. b) Frequency-dependent oscillatory shear experimental data measured at 0.1% strain amplitude. Storage moduli measured at 1 rad s^−1^ are 10.7 kPa for P20, 13.0 kPa for P20-H2, and 15.6 kPa for P20-H5. c) Stress-controlled steady state flow experiments to measure dynamic yield stress, defined as the stress at which viscosity decreases to 10% of its plateau value. d) Step-shear experiments wherein hydrogels are subjected to alternating two-minute periods of low (0.1 s^−1^) and high (10 s^−1^) shear rates demonstrate shear-thinning and self-healing behaviors of P-H hydrogels. e) Temperature ramp experiments conducted by measuring storage and loss moduli at a constant frequency of 1 rad s−1 and strain amplitude of 0.1% while lowering temperature from 40 °C to 4 °C at a rate of 2 °C min^−1^ indicate that P20 undergoes a sol-gel transition at 13.8 °C, P20-H2 undergoes a sol-gel transition at 9.8 °C, and P20-H5 does not undergo a sol-gel transition in the tested temperature range. f) Facile injection of P20-H5 hydrogel and resulting rigid and adherent depot. g) Injection forces of P-H hydrogels through a 25-gauge, 5/8 -inch needle (flow rate = 2 mL min^−1^, n = 3).

PF-127/HPMC hydrogels (P-H hydrogels) are formed by physical mixing of PF-127, HPMC, and soluble components (Fig. 2a). PF-127, a triblock copolymer consisting of two hydrophilic polyethylene oxide (PEO) blocks at each end and a hydrophobic polypropylene oxide (PPO) block in the middle, forms micelles in aqueous solutions.^42^ At elevated temperatures, these micelles pack into a jammed network, forming a hydrogel, where the sol-gel transition temperature depends on the polymer concentration.^37^ We kept the concentration of PF-127 constant at 20 wt.% throughout testing to ensure that the hydrogel is in its solid state at room temperature. Cellulose-derived polymers such as HPMC can strengthen PF-127-based networks, perhaps by incorporating into the hydrophobic cores of the micelles and improving interconnectedness. To capture a range of rheological and diffusional phenomena, both of which affect cargo release in hydrogel vaccines, we varied the concentration of HPMC from 0 to 5 wt.%. Gels consisting of *x* wt.% PF-127 and *y* wt.% HPMC are denoted as P*x*-H*y* hydrogels. Phosphate buffered saline (PBS) was used as the aqueous medium for all materials.

Oscillatory frequency sweeps conducted at 0.1% strain amplitude (verified to be in the linear viscoelastic regime through a strain amplitude sweep) from 0.1 rad s−1 to 100 rad s^−1^ demonstrate that P-H hydrogel formulations are solid-like (Gʹ > Gʺ) over the entire range of frequencies tested (Fig. 2b, ESI Fig. S1†). Stress-controlled steady state flow sweeps were performed to determine the dynamic yield stress of P-H hydrogels. Addition of 5 wt.% HPMC to PF-127 caused the yield stress to increase by more than a factor of 2, from 302 Pa to 724 Pa (Fig. 2c). All formulations tested demonstrated high yield stresses well above the expected stress imposed by the subcutaneous space (estimated to be approximately 25 Pa), so they are expected to form solid depots upon subcutaneous administration.^33^

Step-shear tests were performed by subjecting hydrogel samples to alternating periods of high shear rate (10 s^−1^) and low shear rate (0.1 s^−1^). These tests mimic the healing behavior of the hydrogel after a high shear rate (like that imposed during injection through a needle) is removed. All P-H hydrogel formulations tested show shear-thinning and rapid self-healing following the shear rate reduction (Fig. 2d), indicating likelihood to quickly form depots in vivo post-injection. This behavior indicates that mechanical disruption of the gel during injection would not lead to significant burst release of cargo. Temperature ramps were performed by measuring the storage and performed by measuring the storage and loss moduli at a constant frequency of 1 rad s^−1^ and strain amplitude of 0.1% while decreasing the temperature from 40 °C to 4 °C at a constant rate of 2 °C min^−1^. This test was designed to evaluate the mechanical properties of P-H hydrogels across a temperature range encompassing storage conditions (in the fridge at 4 °C), injection conditions (at room temperature at 25 °C), and in the body (at human body temperature at 37 °C). All formulations tested were solid-like (Gʹ > Gʺ and tan(δ) < 1) at room temperature and at human body temperature; however, only the P20-H5 formulation was solid-like at storage temperature (Fig. 2e; ESI Fig. S2†).

As aqueous solutions of PF-127 are known to exhibit shear-thinning behavior, PF-127-based formulations are promising as depot-forming injectable biomaterials.^43^ All P-H hydrogels were comfortable to inject through a needle and formed solid, adherent depots upon injection (Fig. 2f). We further verified the injectability of P-H hydrogels through injection force measurements with 25-gauge, 5/8 -inch needles. While injection force increased noticeably with HPMC concentration, all formulations met the criteria (maximum force of injection < 38 Pa) for clinically relevant injection force (Fig. 2g, ESI Fig. S3†).^44^

### 2.2. Diffusivity of Cargo in P-H Hydrogels and *In Vitro* Release

Sustained delivery of vaccine components through encapsulation in biomaterials increases the duration for which vaccine antigens are available to the lymph nodes, more accurately simulating natural infections and providing stronger immunity.^45^ Hydrogels can release encapsulated cargo through two mechanisms: diffusion of the cargo out of the mesh of the crosslinked network, or erosion of the material itself, carrying cargo with it.

**Figure 3:**
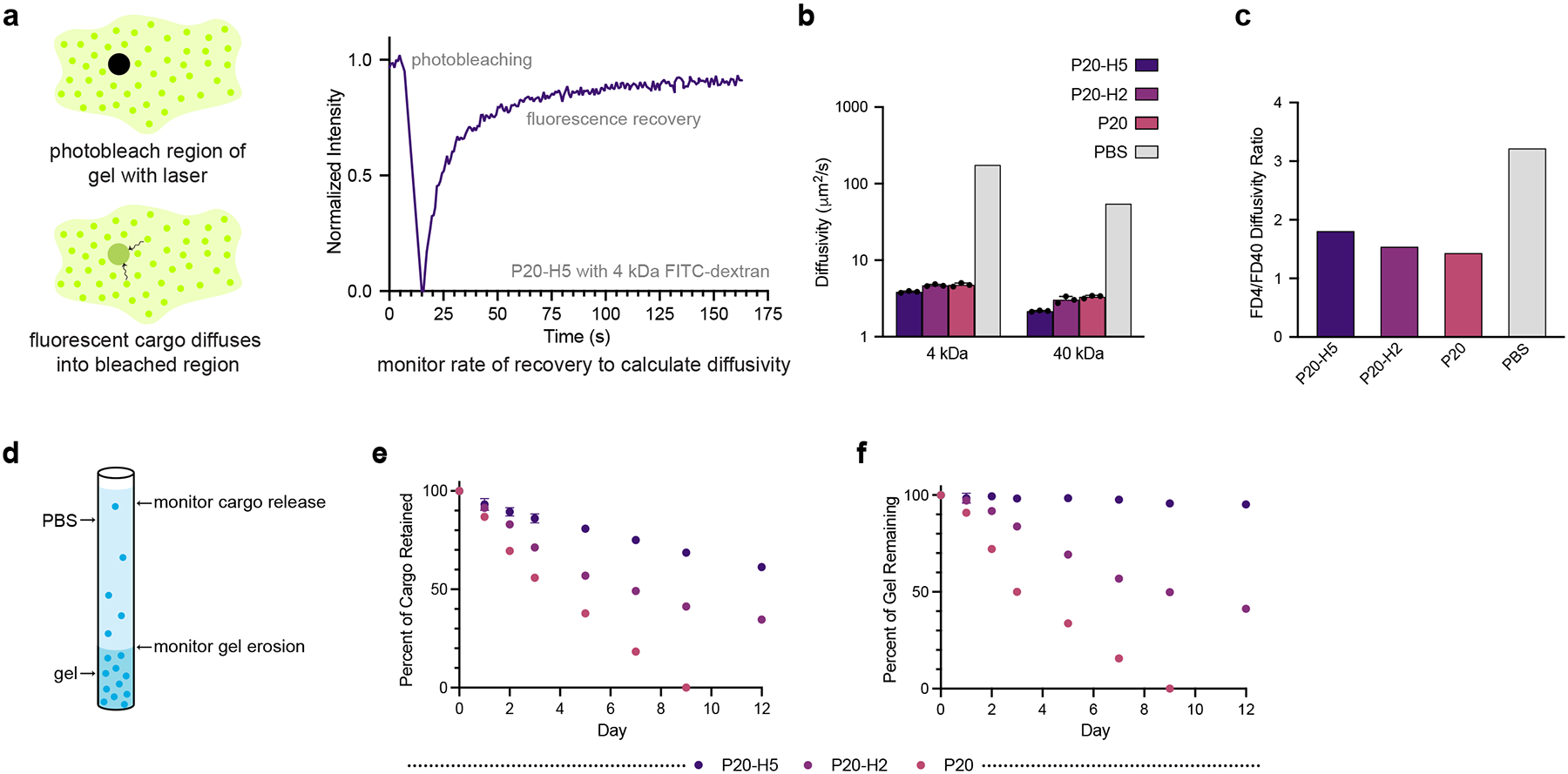
*In vitro* assays depict cargo diffusivity and erosion behavior of P-H hydrogels. a) Schematic of FRAP experiments used to determine diffusivity of fluorescent probes (FD4 and FD40) in P-H hydrogels (n = 3 circular regions per gel). b) Calculated diffusivities for cargo in P-H hydrogels (calculated by fitting FRAP data to a single-phase exponential model) and PBS (calculated via the Stokes-Einstein equation). c) Diffusivity ratio of FD4 to FD40. d) Schematic of in vitro capillary release experiments measuring hydrogel erosion and release of AF647-OVA from P-H hydrogels over time (n = 3). e) Percent AF647-OVA retained in P-H hydrogels over twelve days from in vitro release assays. f) Percent of P-H hydrogel retained over twelve days from *in vitro* release assays.

Fluorescence recovery after photobleaching (FRAP) experiments were performed to calculate diffusivities of variously sized cargo within P-H hydrogels. Fluorescein isothiocyanate-labeled dextran with molecular weight 40 kDa (FD40, R_H_ = 4.5 nm) was chosen to model protein antigens, while fluorescein isothiocyanate-labeled dextran with molecular weight 4 kDa (FD4, R_H_ = 1.4 nm) was chosen to model adjuvants, which are typically smaller molecules. In these experiments, a high intensity laser is applied to a circular region of interest, resulting in photobleaching. The curve of fluorescence recovery over time (Fig. 3a) can be used to calculate the diffusivity of the encapsulated cargo. P-H hydrogels successfully slowed the diffusion of both FD40 and FD4 molecules when compared to phosphate-buffered saline (PBS), in which cargo diffusivity was calculated via the Stokes-Einstein equation (Fig. 3b). In addition, P-H hydrogels present a method for narrowing the gap between the timescale of antigen and adjuvant diffusion, is important for improving immune responses by simultaneously delivering both antigen and adjuvant to the same antigen-presenting cell. 46 The ratio of FD4 to FD40 diffusivity is lower in all P-H hydrogels when compared to PBS, indicating more size-agnostic cargo encapsulation (Fig. 3c).

*In vitro* capillary release assays were performed to monitor cargo release through both hydrogel erosion and diffusion mechanisms when the hydrogel is exposed to aqueous sink conditions at one interface (Fig. 3d). Ovalbumin, Alexa Fluor^®^ 647 Conjugate (AF647-OVA) was loaded into P-H hydrogels and gel was extruded into glass capillary tubes. PBS was added to each tube and replaced at each timepoint, and both the height of the gel (to determine the rate of gel erosion) and the fluorescence of the solvent (to determine cargo release) were recorded. P20-H5 performed significantly better than P20-H2 and P20 in terms of cargo retention, with over 50% of cargo retained by the end of the experiment (Fig. 3e). P20-H5 was also much more resistant to erosion than the other P-H formulations, with over 95% of the gel remaining on day 12 (Fig. 3f). Since P20 fully eroded before the completion of the study, we chose to move forward with P20-H2 and P20-H5 for in vivo experimentation with both model cargo and vaccine cargo.

### 2.3 *In Vivo* Release and Model Vaccine

**Figure 4:**
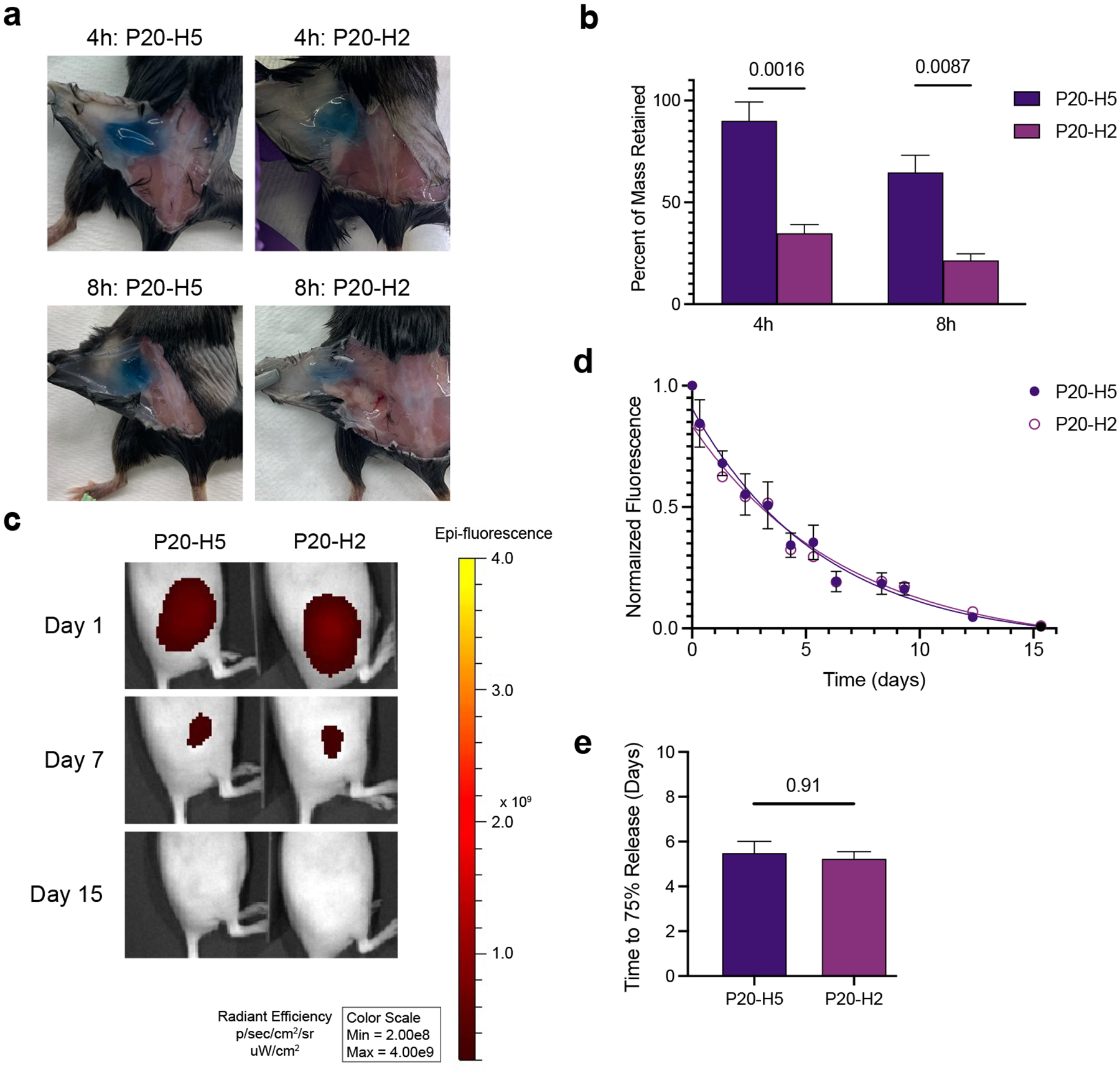
*In vivo* measurements of gel erosion and cargo release. a) Images of P-H hydrogel depots (originally 200 μL injections) 4 and 8 hours post injection. b) Percent mass retained of P-H hydrogels 4 and 8 hours post injection. c) Representative IVIS images of hydrogels loaded with a model vaccine formulation including AF647-OVA in the days after injection. d) Fluorescence intensities over time at the site of injection (normalized to the day 0 fluorescence intensity). e) Time to 75% cargo release for hydrogel vaccines (calculated as twice the half-life extracted from a one-phase exponential decay fit to fluorescence intensity over time data).

As the interface between the hydrogel and the aqueous media is relatively small compared to the total hydrogel volume in the *in vitro* release assays, we expected to see quicker erosion *in vivo* when the entire hydrogel is surrounded by aqueous media in the subcutaneous space. To test this hypothesis, we injected 200 μL of P20-H5 or P20-H2 hydrogel (n = 3–4) into the subcutaneous space on the flanks of C57BL/6 mice, and excised gels at 4 hours and 8 hours post injection (Fig. 4a). Gels were quickly massed post-excision to avoid dehydration, and it was noted that P20-H5 gels have significantly longer in vivo retention at early timepoints than P20-H2 gels (Fig. 4b).

To evaluate longer-term gel degradation in vivo in parallel with vaccine cargo release, we utilized an *In Vivo* Imaging System (IVIS). A sample vaccine formulation containing AF647-OVA and MPLAs was prepared in a 200 μL formulation of either P20-H2 or P20-H5. SKH1-Elite mice (n = 6) were injected subcutaneously on the right flank and were imaged every day for the first week and thrice weekly thereafter until the background fluorescence matched that of the right flank (Fig. 4c). Fluorescent signal for both groups continued to decay until at least day 14, indicating that antigen was continuously released for at least that duration (Fig. 4d). Release on the order of days to weeks has been shown to be an important duration of antigen exposure for vaccines to accurately mimic natural infections, so it is critical that P-H hydrogels meet this threshold.^47^ Release curves and time to 75% cargo release was similar for both P-H hydrogel groups, indicating long-term release kinetics on the order of two weeks (Fig. 4e). Because the burst release kinetics and the long-term release kinetics did not match (i.e. the P20-H2 and P20-H5 gels behaved the same in the long-term release study but differently in the burst release study), we hypothesized that the stretchier skin of hairless SKH1-Elite mice used for IVIS affords a less compressive and fluid-filled subcutaneous space than the thicker skin of C57BL/6 mice used for the burst release study. To test this hypothesis, we repeated the burst release experiment in SKH1-Elite mice and confirmed that the burst release behavior is consistent between both gels (ESI Fig. S4†). Because the short-term mass loss in P20-H5 gels was similar between both strains, we expect the long-term release behavior to be similar in C57BL/6 mice to that displayed in SKH1-E mice.

To compare P-H hydrogels to soluble vaccine formulations as well as more synthetically complex hydrogel-based vaccines, we prepared two additional formulations with AF647-OVA encapsulated in either 100 μL of PNP-1–5 hydrogel (1 wt.% HPMC-C_12_ and 5 wt.% PEG-PLA nanoparticles) or 200 μL of PBS. The volume and formulation of the PNP hydrogel was chosen due to previous demonstrated success in improving humoral response to OVA vaccines.^13^ All hydrogel groups at least doubled the time to 75% release that was observed in PBS (ESI Fig. S5†). In addition, despite faster cargo release, both P-H hydrogels performed similarly to the PNP-1–5 hydrogel in terms of Anti OVA endpoint titers measured over the course of twelve weeks after a prime dose at week 0 and a boost of the same formulations at week 8 (ESI Fig. S6†). ELISpot assays also indicated no significant difference in CD8^+^ T cell response between PNP-1–5 gels and P-H hydrogels (ESI Fig. S7†). It is possible, though difficult to measure, that different P-H hydrogels provide different adjuvanting capabilities despite affording similar release kinetics of protein-sized cargo. Another possibility is that P20-H5 gels afford more extended adjuvant release kinetics than P20-H2 gels in vivo leading to more robust vaccine responses, though this is once again difficult to measure.

### 2.4. SARS-CoV-2 and H5N1 Influenza Vaccines

**Figure 5:**
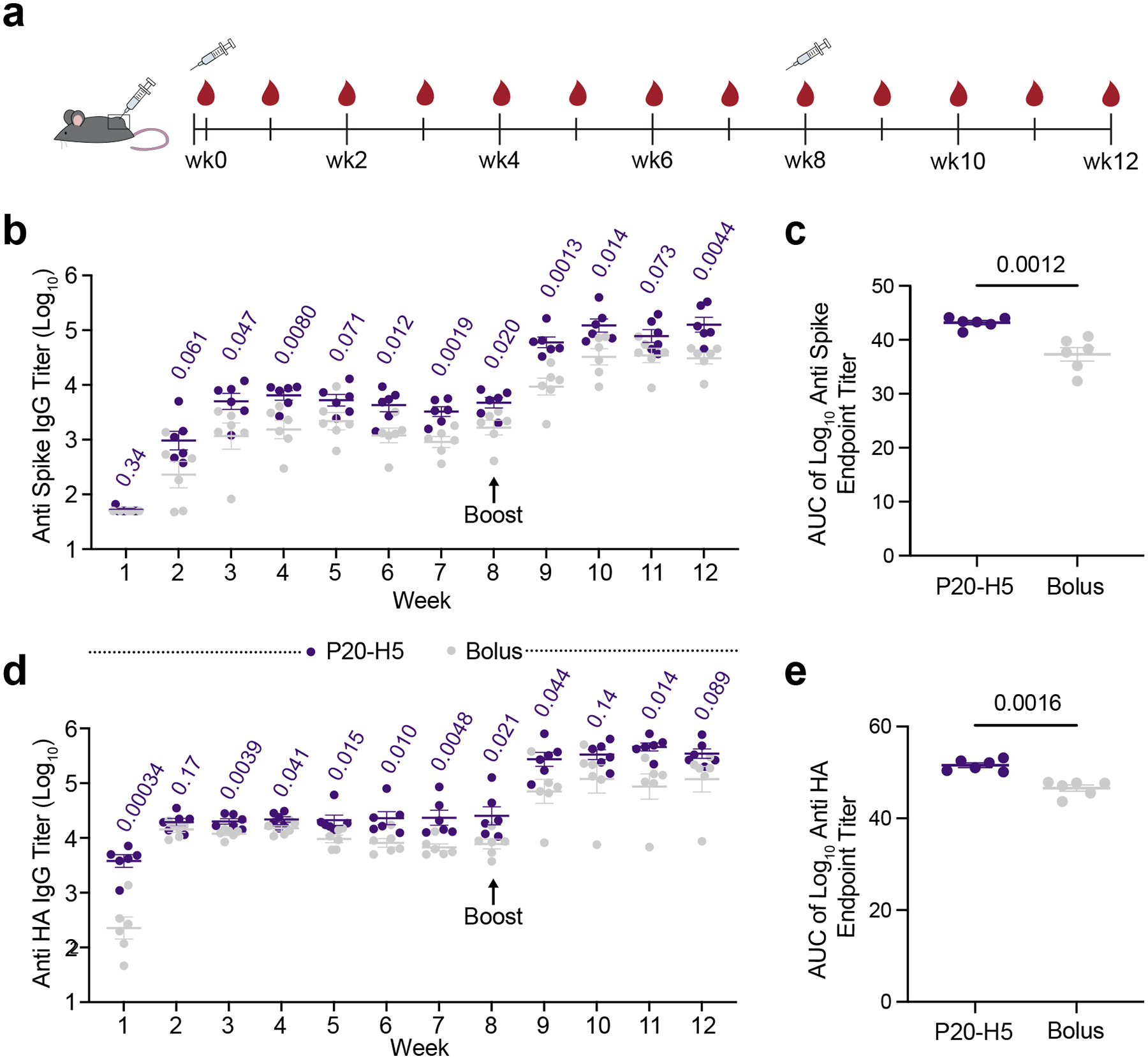
P20-H5 hydrogel vaccines improve humoral response to HexaPro and H5N1 HA-based subunit vaccines relative to emulsion-based formulations. a) Timeline of mouse immunizations and blood collection. Mice were immunized at week 0 and week 8, and serum was collected weekly to determine antigen-specific IgG endpoint titers. b) Anti-Spike IgG titers before and after boosting of P20-H5 and bolus HexaPro vaccines. P values are listed above each timepoint. c) AUC of anti-Spike IgG endpoint titers from week 0 to week 12. d) Anti-H5N1 HA IgG titers before and after boosting of P20-H5 and emulsion-based H5N1 HA vaccines. P values are listed above each timepoint. e) AUC of anti-H5N1 HA IgG endpoint titers from week 0 to week 12.

Following success with a model vaccine formulation, we compared the P-H hydrogel platform to a clinically relevant depot-forming emulsion-based formulation with vaccines against wild-type SARSCoV-2 (WT SARS-CoV-2) and H5N1 influenza. We chose to move forward with the P20-H5 gel, which demonstrated the longest in vitro release profile, the least burst release, and the most robust humoral immune response to the OVA model antigen. In this study, C57BL/6 mice (n = 6) were administered 200 μL injections of either P20-H5 hydrogel-based vaccines or bolus vaccines with a commercial depot-forming adjuvant. Mice were immunized with either wild-type (WT) SARS-CoV-2 HexaPro spike protein (HexaPro), a stabilized version of the WT SARS-CoV-2 spike protein, or Influenza A H5N1 (A/Indonesia/5/2005) hemagglutinin protein (H5N1 HA).^48^ Each P20-H5 hydrogel vaccine was adjuvanted with 3M-052, a small-molecule TLR7/8 agonist, while each emulsion-based bolus vaccine was adjuvanted with AddaVax (an MF59-like squalene emulsion). AddaVax was chosen instead of a bolus formulation containing a TLR agonist due to our previous work demonstrating that there were no significant differences between humoral immune responses in mice receiving a dose of AddaVaxbased and alum/MPLA-based subunit influenza vaccines.^22^ Mice were primed at week 0 of the study and boosted at week 8, and blood was collected weekly for IgG endpoint titer analysis (Fig. 5a).

We evaluated anti-Spike (for WT SARS-CoV-2 vaccines) and anti-H5N1 HA (for H5N1 influenza vaccines) total immunoglobulin G (IgG) titers over twelve weeks using enzyme-linked immunosorbent assays (ELISAs) to measure endpoint titers. For both WT SARS-CoV-2 and H5N1 influenza vaccines, we saw superior humoral immune responses in P20-H5 hydrogel groups characterized by higher antigen-specific IgG titers over the eight-week post-prime period as well as the four-week post-boost period relative to bolus vaccines, with significant differences observed at most timepoints (Fig. 5b, d). In addition, to quantify the potency of antigen-specific IgG responses, we calculated the area under the endpoint titer curves (AUCs). Both P20-H5 hydrogel vaccines resulted in significantly higher AUCs than the corresponding bolus vaccines, indicating that P20-H5 boosted total antibody production of both vaccines (Fig. 5c, e). The P20-H5 hydrogel vaccine against H5N1 influenza also elicits more rapid seroconversion than the corresponding bolus vaccine, illustrated by significant differences in antibody titers at week 1 (p = 0.00034). The increase in antibody titer is not only statistically significant but also indicative of higher immunological potency. For example, at week 12 for the SARS-CoV-2 vaccine, the average IgG titer (raw value) for the P20-H5 group is approximately 160,000 while the average IgG titer for the bolus group is approximately 34,000, representing a 4.7-fold increase. At week 12 for the H5N1 influenza vaccine, the average IgG titer for the P20-H5 group is approximately 386,000 while the average IgG titer for the bolus group is approximately 173,000, representing a 2.2-fold increase.

Durability was assessed by calculating the fold change in antigen-specific IgG from the peak post-prime titer to the minimum post-prime titer following the peak time point. No significant difference in the durability over the first eight weeks was observed for these groups. Future studies could explore extending the boost regimen in order to further evaluate changes in durability (ESI Fig. S8†).

Following the vaccine study, at four weeks post-boost, we assessed antigen-specific IFN-γ^+^ CD8^+^ T cells by ELISpot. Sustained delivery has not proved to significantly affect CD8+ T cell response to vaccines, so we did not expect to see significant differences from ELISpot data.^22^ Surprisingly, we saw a significant improvement in the number of spike-specific IFN-γ^+^ CD8^+^ T cells between the P20-H5 and bolus WT SARS-CoV-2 vaccines (ESI Fig. 9a†). We did not observe a significant difference in the number of HA-specific IFN-γ+ CD8+ T cells between the P20-H5 and bolus H5N1 influenza (ESI Fig. S9b†). We expect that differences are due to the presence of the differing adjuvants (3M-052 vs. AddaVax), as adjuvant selection is known to impact T cell response.^35^

**Figure 6:**
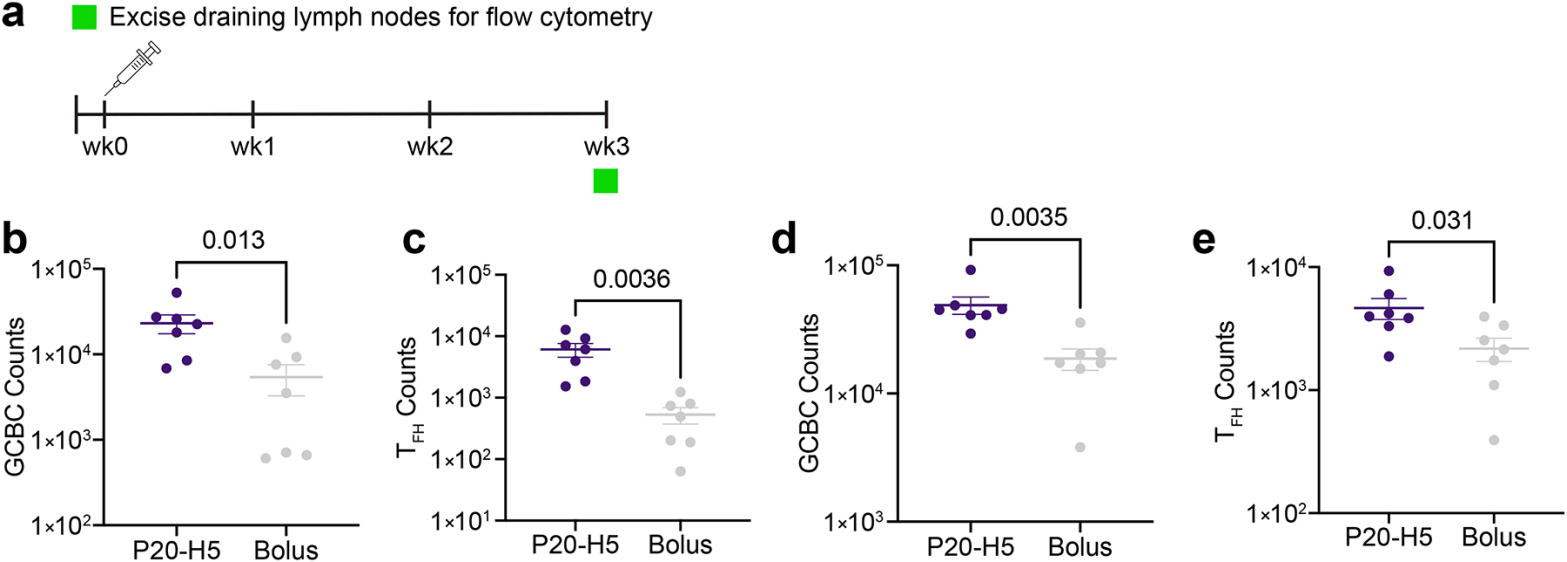
Germinal center responses to P20-H5 and bolus vaccines. a) Mice were immunized with vaccines at week 0 and draining lymph nodes were excised for flow cytometry analysis at week 3. b,c) Count of (b) GCBCs (CD45^+^ CD11b^−^ CD19^+^ CD95^+^ CD38^−^) or (c) T_FH_ cells (CD45^+^ CD11b^−^ CD19^−^ CD3^+^ CD4^+^ CXCR5^+^ PD-1^+^ FOXP3^−^ Bcl6^+^) in dLNs three weeks post-prime immunization with WT SARS-CoV-2 vaccine (n = 7). d, e) Count of (d) GCBCs (CD45^+^ CD11b^−^ CD19^+^ CD95^+^ CD38^−^) or (e) T_FH_ cells (CD45^+^ CD11b^−^ CD19^−^, CD3^+^ CD4^+^ CXCR5^+^ PD-1^+^ FOXP3^−^ Bcl6^+^) in dLNs three weeks post-prime immunization with H5N1 influenza vaccines (n = 7)

To determine the cellular mechanisms for the improvement in humoral response to P20-H5 hydrogel vaccines relative to bolus vaccines, we performed flow cytometry on the draining lymph nodes (dLNs) three weeks post-injection of the same vaccine formulations described earlier in this section (Fig. 6a). The gating strategy used in these studies is shown in ESI Fig. S10†. In mice immunized with WT SARS-CoV-2 vaccines, we observed significant increases in the germinal center B cell (GCBC) counts (p = 0.013) and T follicular helper cell (T_FH_) counts (p = 0.0035) in the P20-H5 hydrogel group relative to the bolus group (Fig. 6b, c). We observed a similar trend in mice immunized with H5N1 influenza vaccines, with significant increases in both GCBC counts (p = 0.0036) and T_FH_ counts (p = 0.031) in the P20-H5 group relative to the bolus group (Fig. 6d, e). Because GCBCs are directly associated with antibody production and T_FH_ cells are involved in GCBC signaling and maturation, the observed elevated counts of these cell types are likely responsible for the improved humoral response from P20-H5 hydrogel vaccines described above. In addition, it has been shown that the magnitudes of the germinal center B cell and T follicular helper cell responses serve as a predictor of long-lasting antibody titers in response to vaccination.^49^ Thus, our data demonstrating higher germinal center B cell and T follicular helper cell counts P-H groups relative to the bolus groups are predictive of more durable responses to P-H vaccines.

## 3. Conclusion

In this work, we developed a simple, scalable, synthesis-free hydrogel platform (P-H hydrogel) prepared by physically mixing commercially available GRAS-listed excipients that enhances the potency of subunit protein-based vaccines. P-H hydrogels are easily injectable due to their shear-thinning behavior and form robust depots in vivo due to their high yield stress and rapid self-healing behavior. Adding HPMC to PF-127-based gels extends the duration of cargo release primarily through preventing rapid hydrogel erosion. This extension of release allows for sustained delivery of protein antigens and adjuvants from the hydrogel depots, leading to enhanced germinal center responses and high antibody titers over the course of twelve-week SARS-CoV-2 and H5N1 influenza vaccine studies. Due to their simple formulation, P-H hydrogels can make sustained vaccine delivery technology more globally accessible, potentially contributing to the eradication of communicable diseases that persist due to insufficiently protective vaccines.

## 4. Experimental

### 4.1. Materials

Pluronic^®^ F-127 (PF-127), hydroxypropyl methylcellulose (HPMC), and bovine serum albumin (BSA) were purchased from Sigma-Aldrich and used as received. Ovalbumin (OVA), OVA 257–264 peptide, MPLAs (Vaccigrade), and AddaVax were purchased from InvivoGen. Ovalbumin, Alexa Fluor^®^ 647 Conjugate (AF647-OVA) was purchased from Thermo Fisher. 3M-052 was purchased from 3M and the Access to Advanced Health Institute (AAHI). Influenza A H5N1 (A/Indonesia/5/2005) Hemagglutinin / HA Protein (His Tag) (H5N1-HA) and SARS-CoV-2 spike protein (40589-V08H4) (spike protein) were purchased from Sino Biological. SARS-CoV-2 HexaPro spike protein was provided by Prof. Neil King at the Institute for Protein Design, University of Washington. Goat anti-mouse IgG Fc secondary antibody horseradish peroxidase (GOXMO) was purchased from Invitrogen. PepMix^®^ SARS-CoV-2 (Spike Glycoprotein) and PepMix^®^ Influenza A (HA/Indonesia (H5N1)) were purchased from JPT. 3,3,5,5”-Tetramethylbenzidine (TMB) ELISA substrate was purchased from Abcam

### 4.2. Preparation of PF-127/HPMC hydrogels (P-H hydrogels)

PF-127 was prepared as a 40 wt.% stock in phosphate-buffered saline (PBS). Air bubbles were removed from the PF-127 stock through centrifugation at 7000 rpm before use. HPMC was prepared as a 20 wt.% stock in PBS at 70 °C, then allowed to gel over the course of an hour in an ice bath. Hydrogel samples were prepared by loading HPMC stock into one syringe and loading PF-127 stock and aqueous components (including vaccine cargo) into a second syringe according to the desired ratio. The syringes were joined through an elbow connector, and components were mixed.

### 4.3. Preparation of polymer-nanoparticle hydrogels (PNP hydrogels)

HPMC-C_12_, PEG-PLA, and PEG-PLA nanoparticles were synthesized as previously reported.^27,50,51^ A PNP hydrogel formulation with 1 wt.% HPMC-C12 and 5 wt.% PEG-PLA nanoparticles was prepared by mixing the corresponding volumes of 6 wt.% HPMC-C_12_ solution and 20 wt.% PEG-PLA nanoparticle solution with antigen and adjuvant cargo solubilized in PBS and added to the nanoparticle solution. Hydrogels were formed by loading the solutions into syringes and mixing them through an elbow mixer.

### 4.4. Rheological characterization

Rheological characterization was performed using a TA Instruments Discovery HR-2 torque-controlled rheometer fitted with a Peltier stage. Unless otherwise noted, rheological tests were performed using a serrated 20-mm plate geometry at 37 °C with a gap height of 500 μm. Strain amplitude sweep measurements were performed at a frequency of 0.1 rad s^−1^ from 0.01% to 100% strain. Dynamic oscillatory frequency sweep measurements were performed with a strain of 0.1% from 0.1 rad s^−1^ to 100 rad s^−1^. Yield stress was calculated from stress-controlled flow sweep measurements, defined as the minimum stress at which the hydrogel’s viscosity drops to one-tenth of its plateau value at low stresses. Temperature ramp measurements were performed at a constant frequency of 1 rad s^−1^ and strain amplitude 0.1%, varying the temperature from 40 °C to 4 °C at a constant rate of −2 °C min^−1^.

### 4.5. Injection force measurements

The instrument setup for injection force measurements was reported previously.^52^ Briefly, a force sensor consisting of a load cell (FUTEK 50 lb load cell, LLB250) connected to a syringe pump (KDS Legato 100) was built. The load cell was calibrated prior to measurements. An individual measurement was performed by loading a 1 mL Air-Tite Luer Lock syringe containing the hydrogel with a 21-gauge, 1-inch needle (BD 305165 PrecisionGlide) attached into the syringe pump and adjusting the dimensions in the software to match that of the syringe. Flow rate was set to 2 mL min^−1^ and injection volume was set to 150 μL. Injection forces over time were recorded by the LabVIEW software. Two consecutive measurements were performed. Injection force was quantified by subtracting the maximum force obtained by the baseline force and converting from kilograms to Newtons.

### 4.6. FRAP measurements and analysis

P-H hydrogels were prepared with the soluble fraction containing either 4 kDa or 40 kDa FITC-Dextran (Sigma-Aldrich). Approximately 100 μL of gel was placed onto a glass slide and imaged using a confocal LSM780 microscope. A high intensity laser (488 nm) was applied to a circular region of interest (ω (radius) = 12.5 μm, n = 3 regions per gel) to bleach it, then fluorescence recovery data was recorded for 200 seconds. Normalized recovery data was fit to a single-phase exponential model and diffusivity was calculated according to the following equation:^53^

$$D=\gamma_{D}\left(\frac{\omega^{2}}{4\tau_{1/2}}\right)$$

Here, γ_*D*_ = τ_1/2_/τ_*D*_, where τ_1/2_ is the recovery half-life (obtained from the fit) and τ_*D*_ is the characteristic diffusion time (obtained from the ZEN software). Diffusivities of fluorescent probes in PBS were calculated using the Stokes-Einstein law of diffusion:

$$D=\frac{k_{B}T}{6\pi \eta R}$$

Here, hydrodynamic radii of 4 kDa FITC-Dextran (R = 0.7 nm) and 40 kDa FITC-Dextran (R = 2.3 nm) were taken directly from Sigma-Aldrich, and the viscosity of PBS (η) was taken to be 0.89 mPa-s.

### 4.7. *In vitro* capillary release studies

P-H gels were prepared, with the soluble fraction containing AF647-OVA. Borosilicate glass capillary tubes (2.7 mm ID) were cut to 4 inches and sealed at one end with epoxy. 100 μL of P-H gel was extruded into the bottom of each tube, and tubes were centrifuged at 1000 rpm to remove air bubbles or pockets. 300 μL of PBS was added to each tube. At each timepoint, 300 μL of PBS was removed and replaced with fresh PBS. The removed PBS was then subjected to fluorescence measurements (640 nm excitation, 670 nm emission) to determine the amount of AF647-OVA released. Fluorescent cargo release was fit to a standard curve, which was calculated by dissolving equivalent gels in different volumes of PBS and performing fluorescence measurements. Between timepoints, capillaries were placed at 37 °C and covered with parafilm to limit evaporation.

### 4.8. Animal protocol

All mice were purchased from Charles River and housed at Stanford University. Animal studies followed National Institutes of Health guidelines and were performed with the approval of the Stanford Administrative Panel on Laboratory Animal Care (APLAC-32109).

### 4.9. *In vivo* burst release studies

Six-week-old female C57BL/6 mice or ten week old female SKH1-Elite mice received a subcutaneous injection of either P20-H2 gel or P20-H5 gel on both flanks with a 21-gauge needle under brief isoflurane anesthesia. Methylene blue was added to the soluble fraction for easier visualization. 4 hours and 8 hours after injection, the mice were euthanized by CO_2_, and hydrogel depots were excised and placed into microcentrifuge tubes. Masses of the depots were calculated by subtracting the previously determined mass of each microcentrifuge tube from the total mass.

### 4.10. IVIS measurements and analysis

Six-week-old female SKH1-Elite mice received a subcutaneous injection of gel on the right flank with a 21-gauge needle under brief isoflurane anesthesia. The injection consisted of either 200 μL of P-H hydrogel vaccine (P20-H5 or P20-H2, containing 98 μg OVA, 2 μg AF647-OVA, and 20 μg of MPLAs) or 100 μL of PNP hydrogel vaccine (containing 1 wt.% HPMC-C_12_, 5 wt.% PEG-PLA nanoparticles, 98 μg OVA, 2 μg AF647-OVA, and 20 μg of MPLAs). Images were collected using the In Vivo Imaging System (IVIS Lago) with an exposure time of 1 s (PNP hydrogels) or 5 s (P-H hydrogels), excitation wavelength of 600 nm, and emission wavelength of 670 nm (binning: medium, F/stop: 1.2). Radiant efficiency measurements were collected with a constant circular region of interest and normalized to the first measurement (collected at time 0, defined as 16 hours post-injection). GraphPad Prism was used to fit single-phase decay models with the initial signal constrained to the time 0 fluorescence value (n = 6). Time to 75% release was calculated as twice the half-life extracted from the fit.

### 4.11. Vaccine formulations

OVA vaccines contained 98 μg of OVA, 2 μg of AF647-OVA, and 20 μg of MPLAs per dose. P-H gels were administered as 200 μL injections, and PNP gels were administered as 100 μL injections. H5N1 influenza vaccines contained 5 μg of H5N1-HA. P20-H5 H5N1 influenza vaccines contained 1 μg of 3M-052, while bolus H5N1 vaccines contained 100 μL of AddaVax. SARS-CoV-2 vaccines contained 0.5 μg of HexaPro. P20-H5 SARS-CoV-2 vaccines contained 1 μg of 3M-052, while bolus SARS-CoV-2 vaccines contained 100 μL of AddaVax. All H5N1 influenza and SARS-CoV-2 vaccines were prepared in PBS to a volume of 200 μL per dose.

### 4.12. Vaccinations

Six-week-old female C57BL/6 mice received a 200 μL subcutaneous injection of either gel or bolus vaccine on the right flank with a 21-gauge needle (for gel) or a 26-gauge needle (for bolus) under brief isoflurane anesthesia. Mice were boosted with the same formulations at week 8.

### 4.13. Mouse serum ELISAs

Antigen-specific IgG endpoint titers in mouse serum were measured with ELISA. MaxiSorp plates (Invitrogen) were coated with OVA (5 μg mL^−1^), H5N1-HA (4 μg mL^−1^), or spike protein (2 μg mL^−1^) overnight at 4 °C and stored at −80 °C for long term storage. The rest of the assay was completed at 25 °C, and plates were washed four times between each step with 0.05 wt.% Tween-20 in PBS. Plates were blocked with a solution of 1 wt.% BSA in PBS (assay buffer). Serum samples were diluted in assay buffer and incubated in the plate for two hours. GOXMO was diluted in assay buffer 1:10000 and incubated in the plate for one hour. TMB substrate was used to develop the plates between 6–10 minutes (kept consistent for a given antigen) and the reaction was stopped with 1N HCl. Absorbance was read at 450 nm, and curves were fit to a three-parameter inhibitor vs. response model in GraphPad Prism. Endpoint dilution titers were defined as the highest-fold dilution at which there is detectable signal.

### 4.14. ELISpot

A Mouse IFN-γ Single-Color ELISpot kit (CTL ImmunoSpot) was used to determine the frequency of antigen-specific IFN-γ producing CD8^+^ T cells. Mice were euthanized by CO_2_, and splenocytes were harvested four weeks post-boost after each vaccine study. 1,000,000 splenocytes (HexaPro study), 500,000 splenocytes (H5N1-HA study), or 400,000 splenocytes (OVA study) per sample were plated into each well and stimulated with 1 μg mL^−1^ peptides from the corresponding antigen (InvivoGen, JPT) for 24 hours at 37 °C. The rest of the assay was conducted following the manufacturer’s instructions.

### 4.15 Flow cytometry of lymph nodes

Mice were euthanized by CO2, and dLNs were harvested at three weeks post-prime and placed into tubes with 1 mL fluorescenceactivated cell sorting (FACS) buffer (PBS, 1 wt.% BSA, 1 mM EDTA) on ice. LNs were dissociated by rubbing between glass slides, passed through 70 μm cell strainers (Celltreat, 229484), and counted. 2 million live cells per sample (or all cells, if less than 2 million were available) were transferred to a 96-well V-bottom plate for staining. All centrifugation steps in the staining protocol were performed at 500 rcf for 5 minutes. Samples were stained with 100 μL of LIVE/DEADTM Fixable Blue Dead (1:800 dilution; Thermo Fisher Scientific, L23105) and incubated for 20 minutes on ice before quenching with 200 μL of FACS buffer and centrifuging. After decanting the supernatant, cells were resuspended in 50 μL of TruStain FcXTM PLUS (anti-mouse CD16/32; 1:50 dilution; BioLegend, 156603) and incubated for 10 minutes on ice.

During incubation, a surface antibody stain was prepared consisting of the following dissolved in FACS buffer: Brilliant Stain Buffer Plus (BD 566385), BUV737 anti-mouse CD19 (1:800 dilution; BD, 612782), APC/FireTM 750 anti-mouse CD8a (1:800 dilution; BioLegend, 100765), APC-Fire 810 anti-mouse CD38 (1:400 dilution; BioLegend, 102745), Alexa Fluor^®^ 532 anti-mouse CD11b (1:400 dilution; Thermo Fisher Scientific, 58-0112-82), PerCP anti-mouse I-A/I-E (MHC II; 1:400 dilution; BioLegend, 107623), PE/DazzleTM 594 anti-mouse CD185 (CXCR5; 1:200 dilution; BioLegend, 145521), BV711 anti-mouse CD4 (1:200 dilution; BioLegend, 100447), PE/Cyanine7 anti-mouse CD95 (1:200 dilution; BD, 557653), BV480 anti-mouse CD279 (PD-1; 1:100 dilution; BD, 746784), Spark NIRTM 685 antimouse CD45 (1:100 dilution; BioLegend, 103167), and BUV496 antimouse CD3 (1:100 dilution; BD, 612955). Without decanting the supernatant, 50 μL of surface antibody stain was added to each well. After incubating for 30 minutes on ice, samples were quenched with 180 μL FACS buffer, and a subsequent wash with FACS buffer was performed.

Fixation and permeabilization buffers were prepared according to manufacturer’s instructions (BD, 554714). Cells were centrifuged and fixed with 200 μL of fixation buffer, incubated at room temperature for 30 minutes, then centrifuged and resuspended in 200 μL of permeabilization buffer. An intracellular antibody stain was prepared consisting of the following dissolved in permeabilization buffer: Alexa Fluor^®^ 488 anti-mouse FOXP3 (1:200 dilution; Thermo Fisher Scientific, 53-5773-82) and Alexa Fluor^®^ 647 anti-mouse Bcl-6 (BD, 561525). Cells were centrifuged, then resuspended in 50 μL of permeabilization buffer, after which 50 μL of the intracellular antibody stain was added to each well without washing. After incubating for 30 minutes at room temperature, samples were quenched with 180 μL permeabilization buffer, then washed with permeabilization buffer. Samples were resuspended in FACS buffer before running on the Cytek^®^ Aurora flow cytometer in the Stanford Shared FACS Facility. FlowJo was used to analyze data, and single antibody cell controls, bead controls, and fluorescence minus one (FMO) controls were used for gating.

### 4.16 Statistical analysis

All results are expressed as mean ± standard error of the mean (SEM). Spread is not shown for ratios of correlated random variables due to lack of a closed form equation. Two-tailed Student’s t-tests were used to compare two groups. One-way analysis of variance (ANOVA) with Tukey’s multiple comparison tests were used to compare across multiple groups. Selected p-values are shown in the text and the Supporting Information. Results were accepted as significant if p < 0.05. GraphPad Prism 10 was used to perform statistical analysis.

## Supplementary Material

Supplementary Information

Supplementary Information available: [details of any supplementary information available should be included here].

## Data Availability

All data reported in the paper are available from the corresponding author upon reasonable request.
