# Supplementary material for: Enabling Global Access to Potent Subunit Vaccines with a Simple and Scalable Injectable Hydrogel Platform
Source: Biomater Sci. Author manuscript; Available in PMC 2026 Jan 25. (PMC12832181; doi:10.1039/d5bm01131k)
Supplement: Supplementary Information [file NIHMS2129262-supplement-Supplementary_Information.pdf]

Supporting Information

Enabling Global Access to Potent Subunit Vaccines with a Simple and Scalable Injectable Hydrogel Platform

Priya Ganesh[1], Alexander N. Prossnitz[1], Carolyn K. Jons[1], Noah Eckman[2], Alakesh Alakesh[1], Ye Eun Song[1], Samya Sen[1], Eric A. Appel[,1,3,4,5,6]*

1. Department of Materials Science and Engineering, Stanford University, Stanford, CA 94305, USA
2. Department of Chemical Engineering, Stanford University, Stanford, CA 94305, USA
3. Department of Bioengineering, Stanford University, Stanford, CA 94305, USA
4. Sarafan ChEM-H Institute, Stanford University, Stanford, CA 94305, USA
5. Department of Pediatrics – Endocrinology, Stanford University, Stanford, CA 94305, USA
6. Woods Institute for the Environment, Stanford University, Stanford, CA 94305, USA

E-mail: eappel@stanford.edu

Table of Contents

Supplemental Figure 1: Strain amplitude sweeps of P-H hydrogels 2
Supplemental Figure 2: Tan delta from temperature ramp experiments.................................. 2
Supplemental Figure 3: Force vs. time curves for injection force experiments........................ 3
Supplemental Figure 4: Percent mass remaining in SKH1-Elite mice 3
Supplemental Figure 5: Time to 75% release of AF647-OVA 4
Supplemental Figure 6: Humoral response to OVA vaccines.. 4
Supplemental Figure 7: $CD8^+$ T cell response to OVA vaccines.. 5
Supplemental Figure 8: Post-prime durability of SARS-CoV-2 and influenza vaccines 6
Supplemental Figure 9: $CD8^+$ T cell response to SARS-CoV-2 and influenza vaccines 6
Supplemental Figure 10: Gating strategy for flow cytometry experiments 7

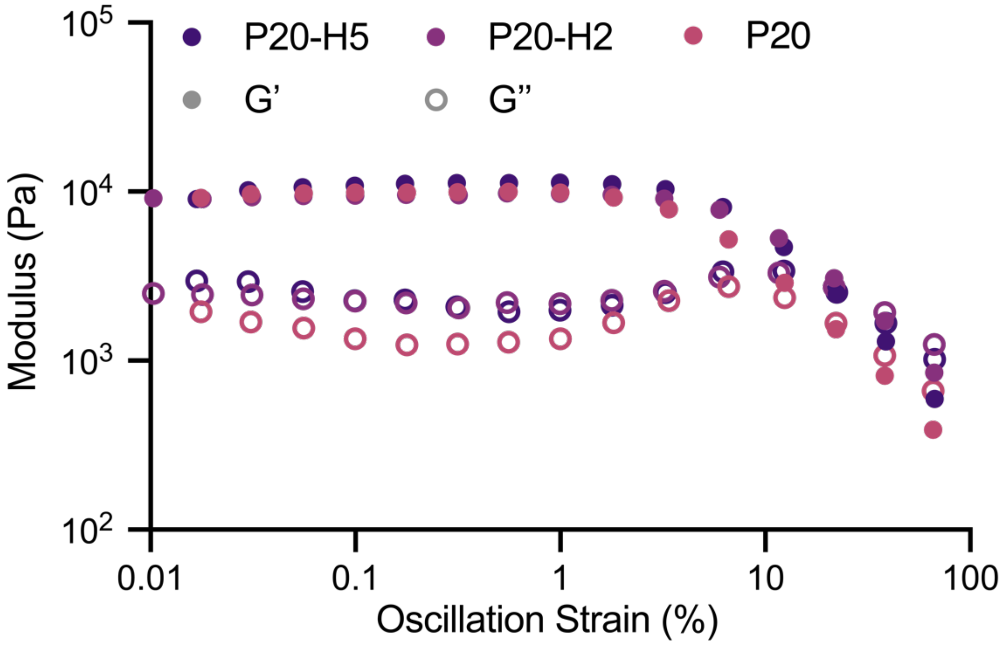

Figure S1. Strain amplitude sweep conducted at 1 rad s^{-1} demonstrates linear viscoelastic regime of gels up to 1% strain.

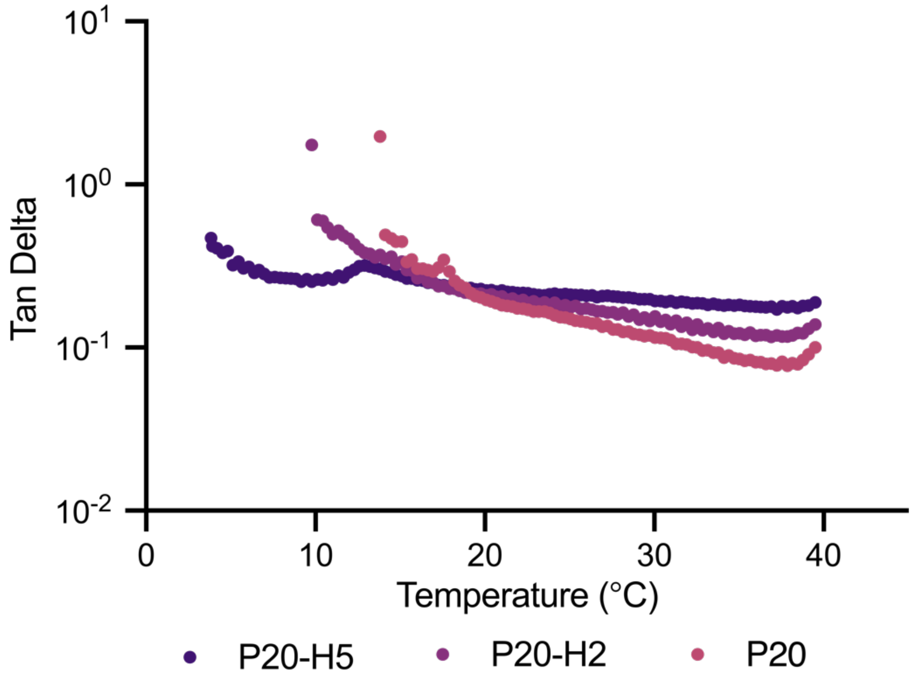

Figure S2. Tan delta (G''/G') measured from temperature ramp experiments conducted by measuring storage and loss moduli at a constant frequency of 1 rad s^{-1} while lowering temperature from 40ºC to 4ºC at a rate of 2ºC min^{-1}. Tan delta = 1 marks the sol-gel transition.

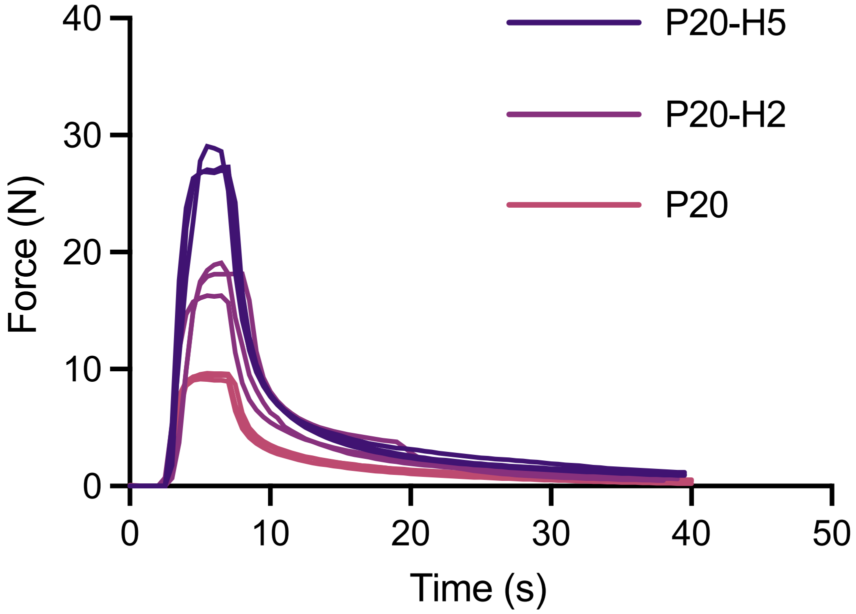

Figure S3. Force vs. time curves for P-H hydrogels over the course of a 150 μL injection at 2 mL min^{-1}. Initial force plateau is subtracted from each curve to reach a starting value of 0 N.

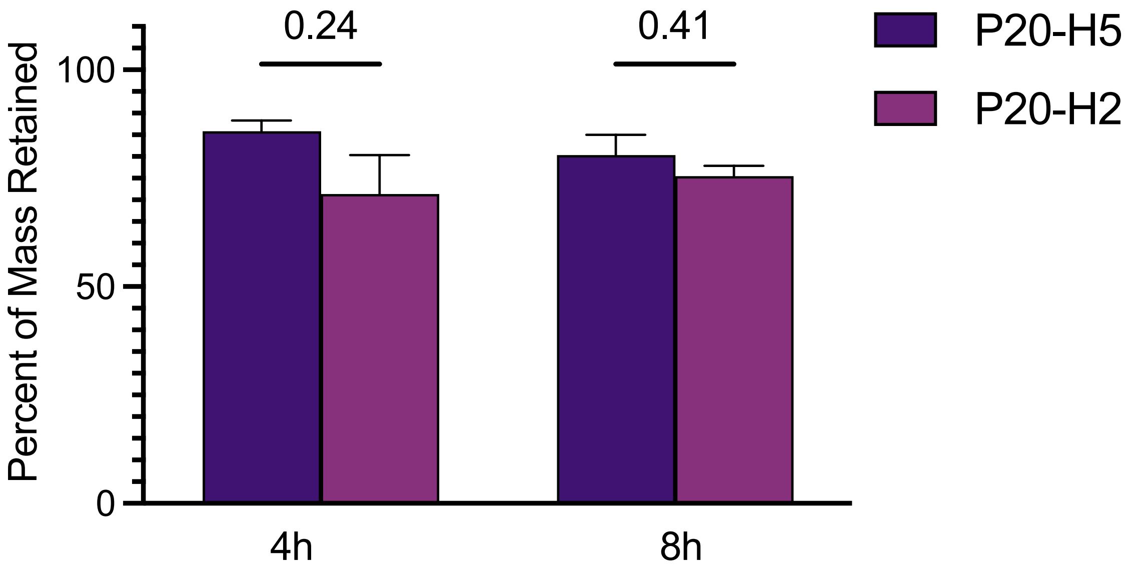

Figure S4. Percent mass retained of P-H hydrogels 4 and 8 hours post injection after 200 μL injection in SKH1-Elite mice (n = 3).

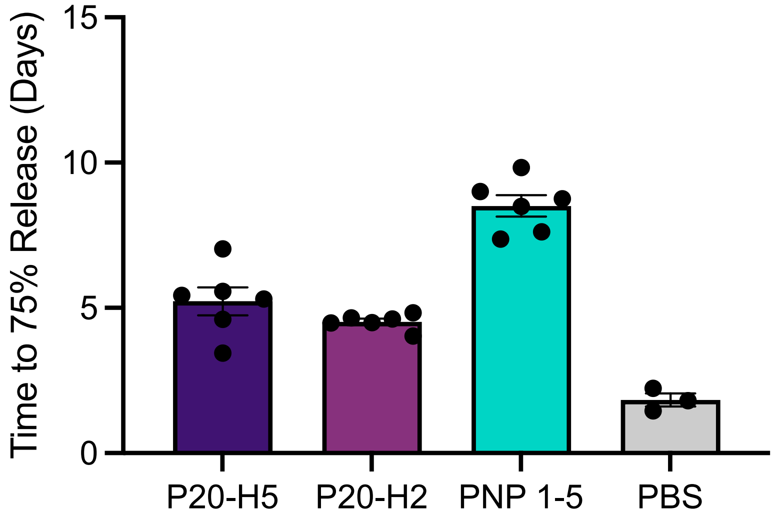

Figure S5. Time to 75% AF647-OVA release for hydrogels (200 µL for P-H hydrogels, 100 µL for PNP 1-5 gel, calculated as twice the half-life extracted from a one-phase exponential decay fit to fluorescence intensity over time data) as compared to 200 µL PBS. All formulations contained 2 µg AF647-OVA. P20-H5, P20-H2, and PNP 1-5 formulations contained an additional 98 µg OVA and 20 µg MPLAs.

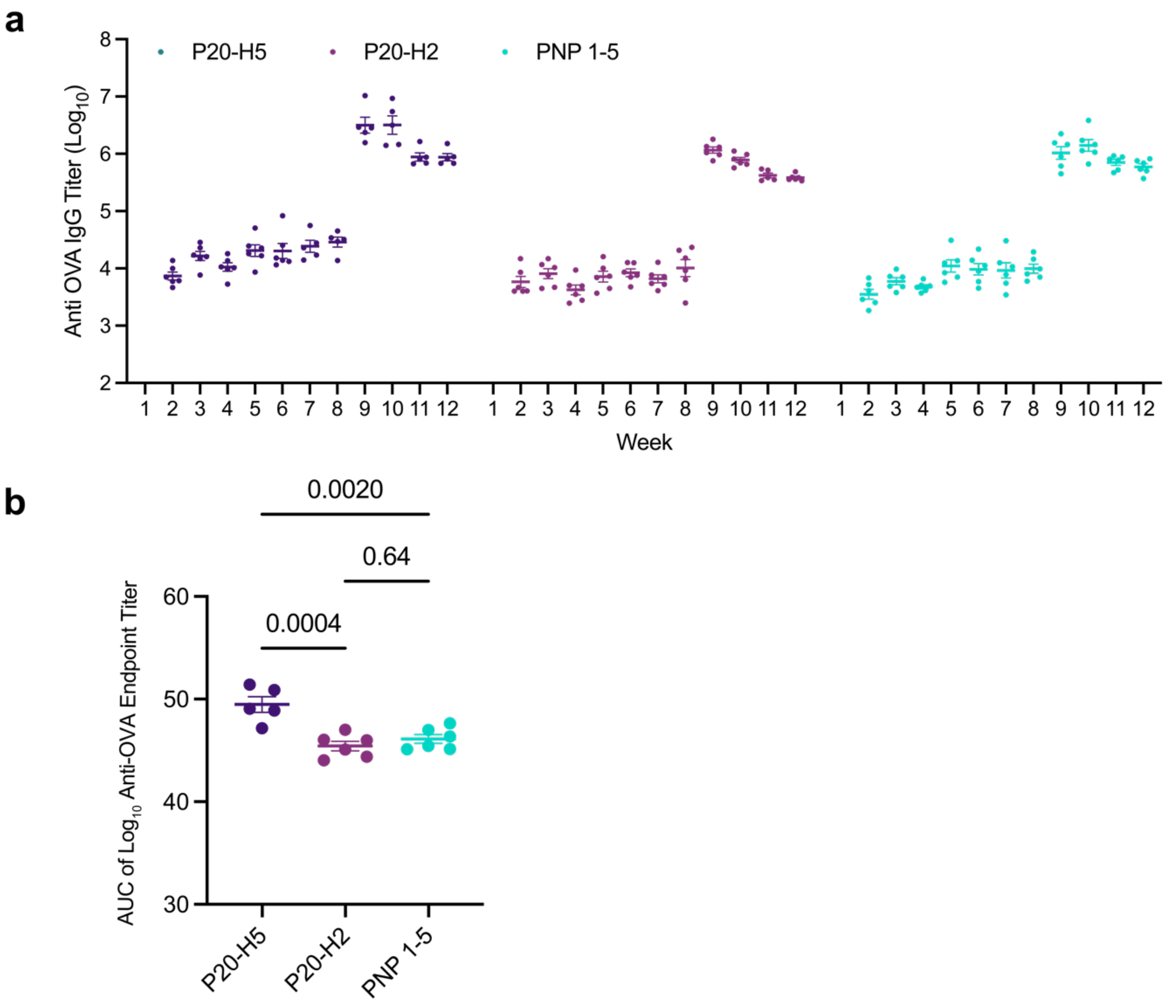

Figure S6. Humoral response to OVA vaccines. a) Anti-OVA IgG titers before and after boosting of hydrogel vaccines. b) AUC of anti-OVA IgG endpoint titers from week 0 to week 12.

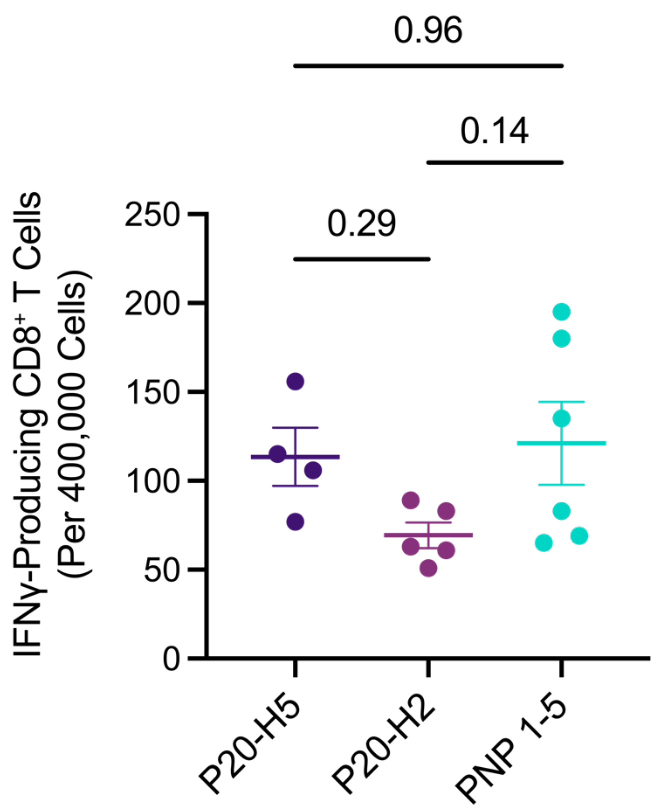

Figure S7. Count of IFNγ producing CD8$^+$ T cells per 400,000 splenocytes after prime-boost immunization with OVA vaccine and stimulation with SIINFEKL peptide.

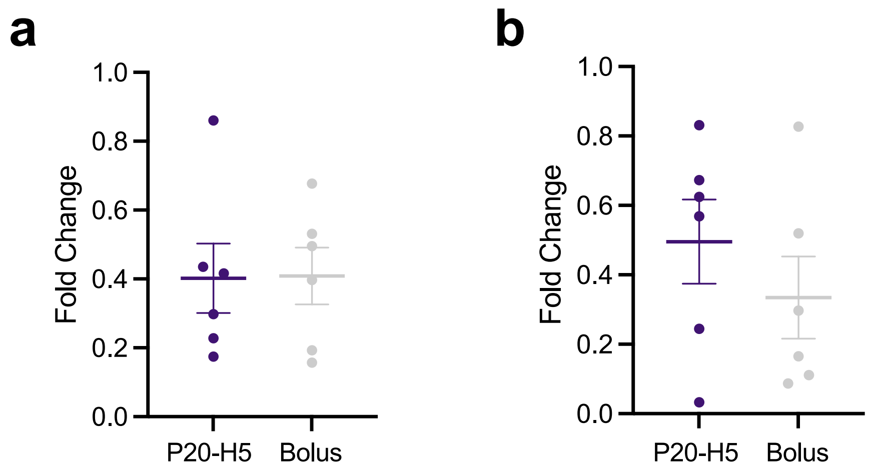

Figure S8. Post-prime durability of (a) SARS-CoV-2 vaccines and (b) H5N1 influenza vaccines, measured as a fold change from peak post-prime titer to minimum post-prime titer following the peak.

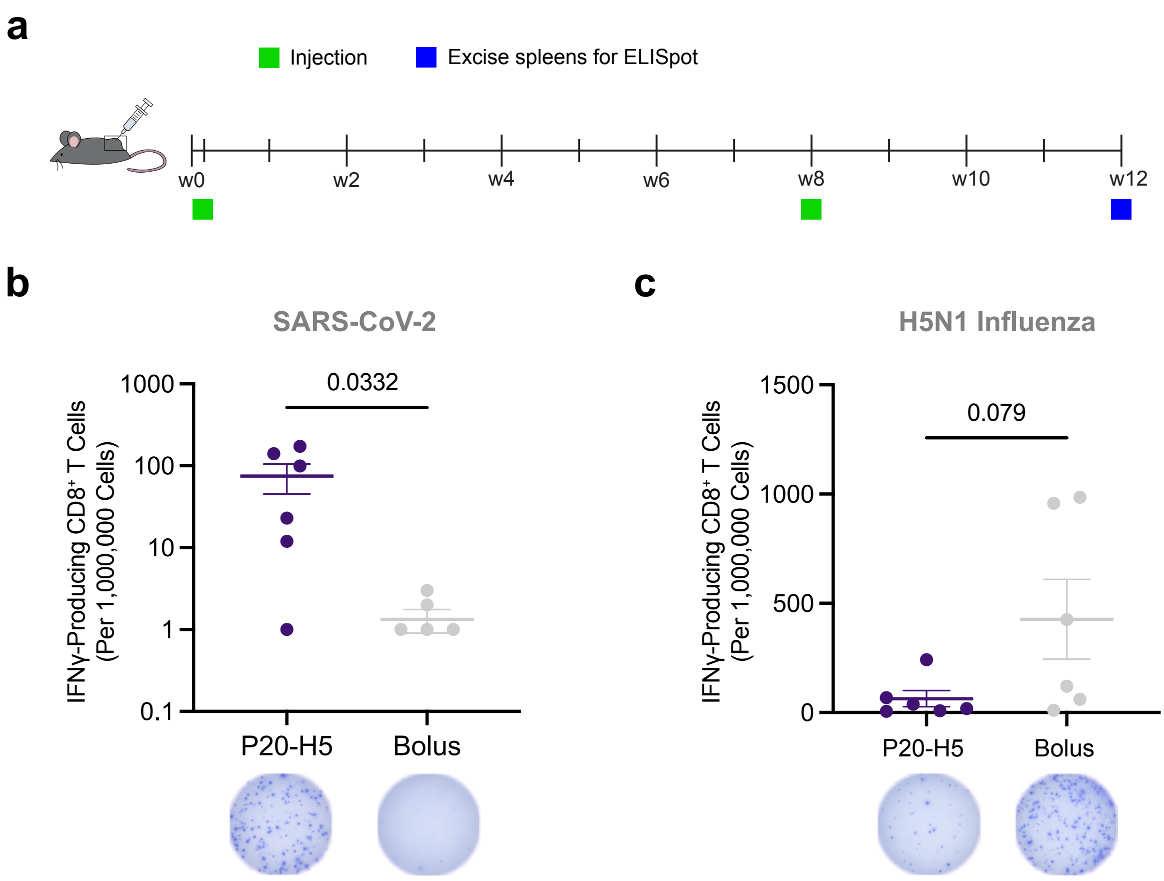

Figure S9. T cell responses to SARS-CoV-2 and H5N1 influenza vaccines. a) Timeline of injections. Gel and bolus vaccines (formulated as in antibody titer experiments) were administered to mice at weeks 0 and 8 of the study. Mice were euthanized at week 12 and spleens were excised and processed for ELISpot. b) Count of IFNγ producing $CD8^{+}$ T cells per 1,000,000 splenocytes after prime-boost immunization with SARS-CoV-2 vaccine and stimulation with spike peptide pool. c) Count of IFNγ producing $CD8^{+}$ T cells per 500,000 splenocytes after prime-boost immunization with H5N1 influenza vaccine and stimulation with H5N1 hemagglutinin peptide pool.

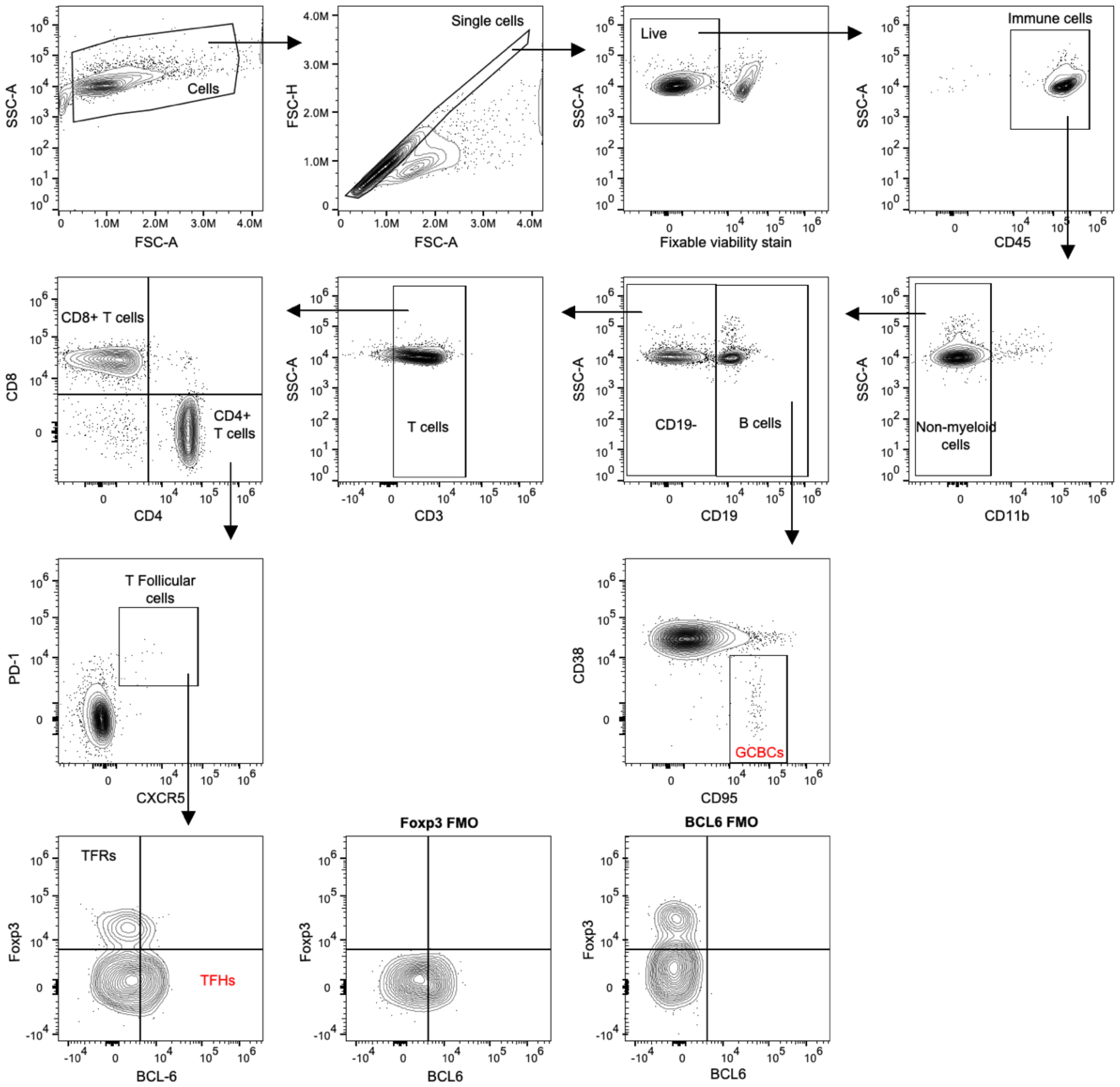

Figure S10. Gating strategy for flow cytometry on lymph nodes three weeks post vaccine administration. A representative sample is shown along with fluorescence minus one (FMO) controls where needed.
